# Degree of amyloid-β burden could be indicative of the primary etiology underlying dementia

**DOI:** 10.1007/s00259-024-06875-8

**Published:** 2024-08-30

**Authors:** Lyduine E. Collij, Adrian Smith, Christopher Buckley

**Affiliations:** 1 Clinical Memory Research Unit, Department of Clinical Sciences Malmö, Faculty of Medicine, Lund University, Lund, Sweden; 2 Radiology and Nuclear Medicine, Amsterdam UMC, location VUmc, Amsterdam, the Netherlands; 3 GE Healthcare, Amersham, United Kingdom

Traditionally, amyloid positron emission tomography (PET) imaging is reduced to a binary measure of normal or abnormal to indicate the absence or presence of amyloid-β (Aβ) pathology. This rather straightforward approach has shown high clinical utility, leading to a change in patient diagnosis and management in a significant percentage of the clinical population [1, 2]. However, recent studies have suggested the value of capturing the degree of Aβ burden, either through visual read [3] or quantification [4], to determine whether the observed extent of pathology was expected based on the clinical stage [5]. For example, patients with lewy body dementia (DLB) who had concomitant Aβ-pathology, showed to have lower burden on amyloid-PET compared to their AD dementia counterparts [5]. Here we describe the case of the GEHC [^18^F]flutemetamol PET Phase-III pathology study to illustrate this potential future use of amyloid-PET in the clinical routine.

An 81-year-old female clinically diagnosed with severe dementia (MMSE = 0) and Parkinson’s disease was included in the study (post-mortem delay 10.8 months). Neuropathological diagnosis was advanced LB disease, leading to a clinicopathological diagnosis of DLB. Both the amyloid-PET scan and post-mortem assessment were indicative of amyloid pathology, though to a moderate degree. The PET image showed an atypical pattern of uptake, which was predominantly in the posterior regions of the brain.

With the expected arrival of multiple anti-amyloid disease-modifying therapies, accurate diagnosis and determining the presence of co-pathologies will be key to make accurate treatment decisions at the individual level, considering their potential effect on anti-amyloid monoclonal antibodies treatment efficacy [6].

**Figure:**
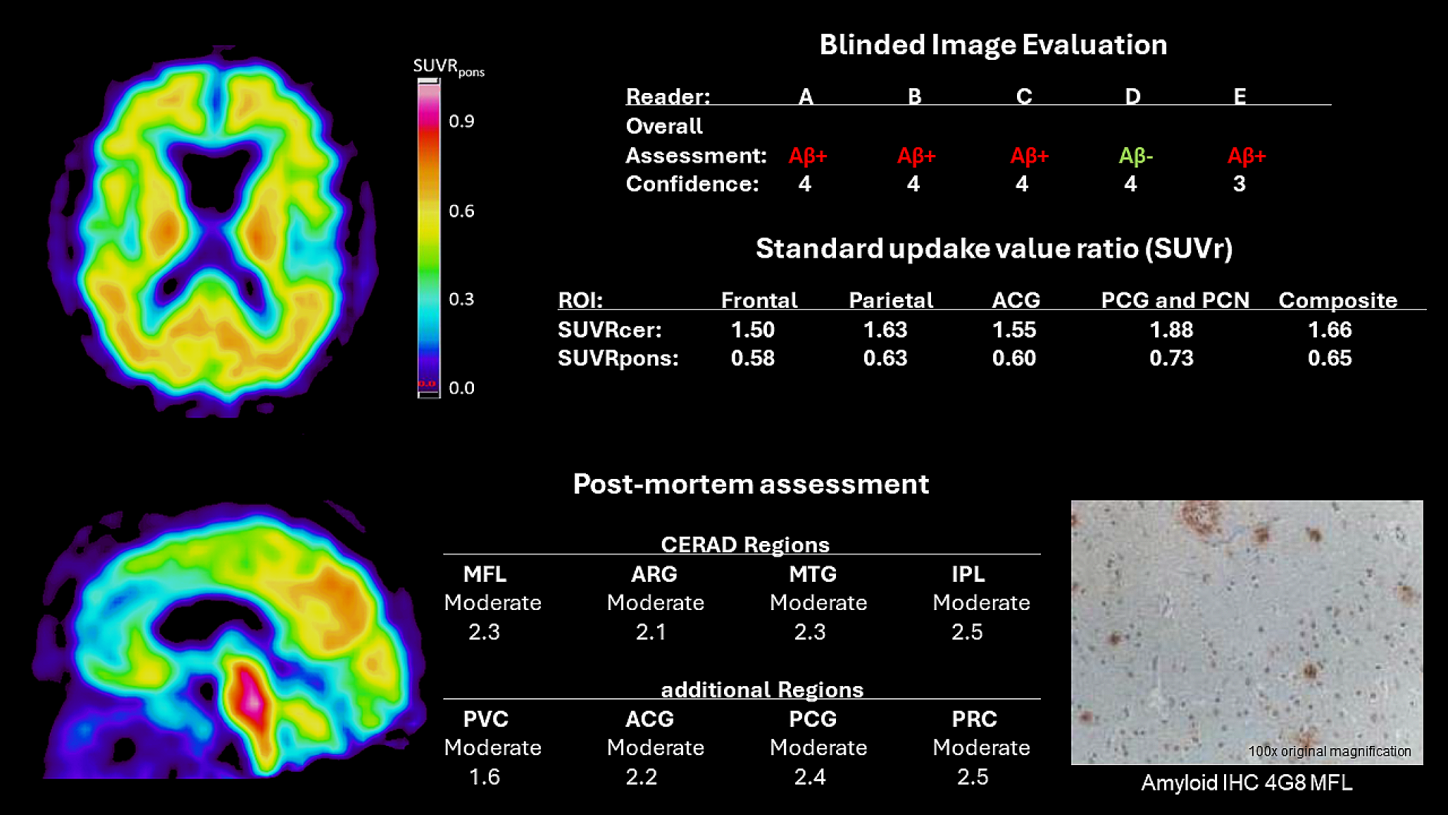
*MFL: midfrontal*,* STG: superior temporal gyrus*,* MTG: middle temporal gyrus*,* IPL: inferior parietal lobe*,* PVC: primary visual cortex*,* ACG: anterior cingulate gyrus*,* PCG: posterior cingulate gyrus*,* PRC/PCN: precuneus, IHC: immunohistochemistry*

## Data Availability

Data is available upon reasonable request with GE Healthcare.

## Declarations

**AS** is a full-time employee of GE Healthcare.

**CB** is a full-time employee GE Healthcare.

### Additional information

For related content on neuroimaging for Alzheimer’s disease, please visit: https://neuroimaging-alzheimers-disease-ime.springermedicine.com/.
